# An innovative ecohealth intervention for Chagas disease vector control in Yucatan, Mexico

**DOI:** 10.1093/trstmh/tru200

**Published:** 2015-01-19

**Authors:** Etienne Waleckx, Javier Camara-Mejia, Maria Jesus Ramirez-Sierra, Vladimir Cruz-Chan, Miguel Rosado-Vallado, Santos Vazquez-Narvaez, Rosario Najera-Vazquez, Sébastien Gourbière, Eric Dumonteil

**Affiliations:** a Laboratorio de Parasitología, Centro de Investigaciones Regionales “Dr. Hideyo Noguchi”, Universidad Autónoma de Yucatán, Mérida, Yucatán, Mexico; b Departamento de Control de Vectores, Servicios de Salud de Yucatán, Mérida, Yucatán, Mexico; c EA 4218 Institut de Modélisation et d'Analyses en Géo-Environnement et Santé, Université de Perpignan Via Domitia, Perpignan, France

**Keywords:** Chagas disease, Community participation, *Triatoma dimidiata*, Vector control

## Abstract

**Background:**

Non-domiciliated (intrusive) triatomine vectors remain a challenge for the sustainability of Chagas disease vector control as these triatomines are able to transiently (re-)infest houses. One of the best-characterized examples is *Triatoma dimidiata* from the Yucatan peninsula, Mexico, where adult insects seasonally infest houses between March and July.

**Methods:**

We focused our study on three rural villages in the state of Yucatan, Mexico, in which we performed a situation analysis as a first step before the implementation of an ecohealth (ecosystem approach to health) vector control intervention.

**Results:**

The identification of the key determinants affecting the transient invasion of human dwellings by *T. dimidiata* was performed by exploring associations between bug presence and qualitative and quantitative variables describing the ecological, biological and social context of the communities. We then used a participatory action research approach for implementation and evaluation of a control strategy based on window insect screens to reduce house infestation by *T. dimidiata*.

**Conclusions:**

This ecohealth approach may represent a valuable alternative to vertically-organized insecticide spraying. Further evaluation may confirm that it is sustainable and provides effective control (in the sense of limiting infestation of human dwellings and vector/human contacts) of intrusive triatomines in the region.

## Introduction

Chagas disease is a major cause of morbidity and mortality in the Americas, with an estimated 9–10 million people currently infected, causing an annual burden of 806 170 disability-adjusted life years (DALYs) and an annual health care cost of US$627.46 million.^1,2^ In Mexico, the Ministry of Health reports a few hundred cases every year,^3^ but estimates suggest that there may currently be up to 6 million people infected.^4^ The only nation-wide studies are from the late 1980s, and indicate a national seroprevalence of 1–2%, with an important heterogeneity among regions and states.^5–7^ In Yucatan, seroprevalence ranges from about 1% in urban areas to 4% in rural villages.^8–10^

In most countries where Chagas disease is endemic, current control of disease transmission is based mostly on empirical insecticide spraying to reduce house infestation by triatomine vectors. However, this vertically-organized approach of vector control is based on a simplistic view focused only on the vectors and not on all the determinants of the disease. In addition, the difficulties associated with the sustainability of this strategy, together with the emergence of insecticide resistance^11^ and recognition of the increased role of intrusive triatomines in parasite transmission to humans, are making long-term vector control challenging.^12,13^ There is thus a strong need for better and integrated tools for the control of Chagas disease, as proposed by the One Health^14–16^ or the ecohealth (ecosystem approach to health)^17–19^ approaches. These approaches are based on the concept that human health cannot be considered in isolation, but is linked to the quality of the environment in which people live and the ecosystem to which they belong.^20^ Thus, ecohealth promotes interventions targeting the multiple determinants of disease transmission in an integrated manner, including biological, ecological and social aspects, for a more effective and sustainable control.^18,19,21,22^ The approach also focuses on the impact of people on ecosystems, and its implications to improve human health and the quality of life, in a sustainable manner. It is based on the use of transdisciplinary participatory research, which may achieve better health outcomes through integrated vector control.^22^ Ecohealth strategies are emerging as more rational, sustainable and cost-effective than vertically-organized and widespread empirical insecticide spraying.^17–19,22–26^

Non-domiciliated (intrusive) triatomines disperse from peridomestic and sylvatic sites to occasionally infest or re-infest houses. One of the best-characterized examples is *Triatoma dimidiata* from the Yucatan peninsula, Mexico, where we have shown that adult *T. dimidiata* transiently infest houses on a seasonal basis during the months of March to July.^27–29^ Population genetics and mathematical models indicate that house infestation is caused by the seasonal dispersal of bugs from both the peridomestic and sylvatic habitats surrounding the villages, while triatomine reproduction in the domestic habitat (i.e. colonization) plays a negligible role.^30–32^

Under the scenario of transient seasonal infestation by *T. dimidiata*, both field and modeling studies have shown that conventional insecticide spraying is of limited efficacy to control domestic infestation.^33,34^ On the other hand, alternative strategies such as the use of insect screens (physical barriers) and long-lasting insecticide impregnated curtains (chemical barriers) were able to reduce house infestation by 80–95% for at least two consecutive years in pilot studies.^34–36^ The cleaning of peridomiciles to reduce bug populations has also been found to reduce *T. dimidiata* abundance inside houses by about 50%, largely because only bugs dispersing from peridomestic colonies are controlled, leaving infestation by sylvatic bugs unaffected.^35,36^ Peridomicile cleaning may nonetheless be a valuable component of integrated vector management.^35,36^ Combining strategies described above may provide an effective and sustainable vector control alternative to insecticide spraying in Yucatan (in the sense of reducing/eliminating vector populations inside human dwellings and of limiting vector/human contacts rather than in killing vector populations). Nonetheless, further transdisciplinary and integrative studies are needed to provide a deeper understanding of the ecological, biological and social (eco-bio-social) determinants for house infestation by *T. dimidiata* and *Trypanosoma cruzi* transmission to humans. Indeed, a limited understanding of the factors driving transient infestation has restricted the design of targeted vector control interventions. In this work, we review studies aimed at identifying the major eco-bio-social determinants contributing to house infestation by intrusive *T. dimidiata*, and the implementation process of an integrated ecohealth intervention tailored to control Chagas disease vectors in Yucatan, Mexico.

## Materials and methods

### Situational analysis of the eco-bio-social determinants of house infestation by *T. dimidiata* (Phase I)

This phase of the project was carried out between early 2011 and mid-2012. We focused our study on three rural villages (Teya, Sudzal and Bokobá) in the state of Yucatan. Typical households in rural Yucatan are composed of four to five individuals who self-identify as Mayan (over 90% are native Mayan speakers). They are mostly subsistence farmers with no education beyond elementary school. Only a minority of the population have formal employment and most receive social welfare benefits. Over the past 15 years government programs have funded the construction of concrete block housing, which has gradually replaced traditional adobe huts. Houses usually consist of two rooms, at least one of which serves as a dedicated bedroom. A cooking area is often located outside. Houses are surrounded by a peridomicile, demarkated by walls made of piled rocks. Most families keep domestic animals in the peridomicile, mainly dogs, chickens, cats and song birds.^37,38^

The identification of the key determinants affecting the presence or absence of *T. dimidiata* in human dwellings was performed by exploring associations between bug presence in houses and a large number of variables describing house environmental factors, as well as the social, cultural and economic context and practices of the inhabitants. Bug presence in houses was monitored by community participation, with households reporting to the village health center any bugs found in their houses. As in previous studies, 15–20% of houses were found to be infested by triatomines.^37^ Data on the habitat, environmental and sociocultural factors, including gender issues, were collected through a variety of complementary methods. Freelisting (i.e. community members were asked to list biting insects and insect control methods) was first used to gain some perspective on community priorities in terms of insect control, followed by ranking exercises, which in turn informed the development of a focus group discussion guide. Results from all previous methods were used to design a survey to quantify and further explore specific aspects. This mixed methods approach allowed us to triangulate complementary data and corroborate conclusions, making maximum use of both qualitative and quantitative approaches.^37–39^ House-level data were georeferenced to produce maps and spatial analyses were performed.

A multi-model approach based on logistic regressions followed by model selection using Akaike information criteria^40^ and model averaging^41^ led to the identification of a limited number of factors that accounted for most of the variability in infestation at the level of groups of houses.

### Implementation and evaluation of the vector control intervention (Phase II)

Effectiveness and sustainability of vector control relies on community members' acceptance and adoption of interventions. Community participation in intervention planning, including decisions about logistics and coordination, are likely to result in vector control programs that are better tailored to community members' needs as well as increased community ownership of the intervention.^42^ In order to optimize the installation of screens as a barrier against house infestation with triatomines, we engaged multiple stakeholders (community members, local government, social workers/leaders, health center, carpenters, research groups) in a participatory planning and implementation process which was developed from late 2012 to mid-2013.

Stakeholders meetings were organized in two of the villages (Teya and Sudzal) to discuss and organize the vector control intervention. First, the results of Phase I were presented. Next, different strategies for implementation were analyzed in an iterative process over several meetings, until the stakeholders reached an agreement on the best implementation strategy. All participants provided written informed consent before engaging in any research activities.

## Results

### Situational analysis of the eco-bio-social determinants of house infestation by *T. dimidiata* (Phase I)

Analysis of the determinants of house infestation revealed that none of the variables related to the socio-economic status of the household, the education level or general cultural practices such as sleeping or cleaning habits of the house were found to be associated with infestation. On the other hand, we found that the presence of dogs, chickens and potential refuges, such as rock piles in the peridomicile, as well as the proximity of houses to vegetation at the periphery of the village and to public light sources, were major risk factors for infestation.^37^ Importantly, these determinants were able to account for most of the variability in infestation at the level of groups of houses (R^2^=0.85), suggesting that we identified the most relevant determinants of infestation and that those we may have missed contributed little.^37^ These results largely confirmed the intrusive nature of *T. dimidiata* in the region, the importance of the peridomicile as a habitat for bugs that infest houses, and the contribution of previously-identified risk factors.^43–45^ The potential role of chickens and dogs in domestic infestation suggested that these determinants may be targeted for improved vector control. For example, improved chicken coops and the removal of triatomines from dog resting areas may contribute to reduced peridomestic infestation.

**Figure 1.**
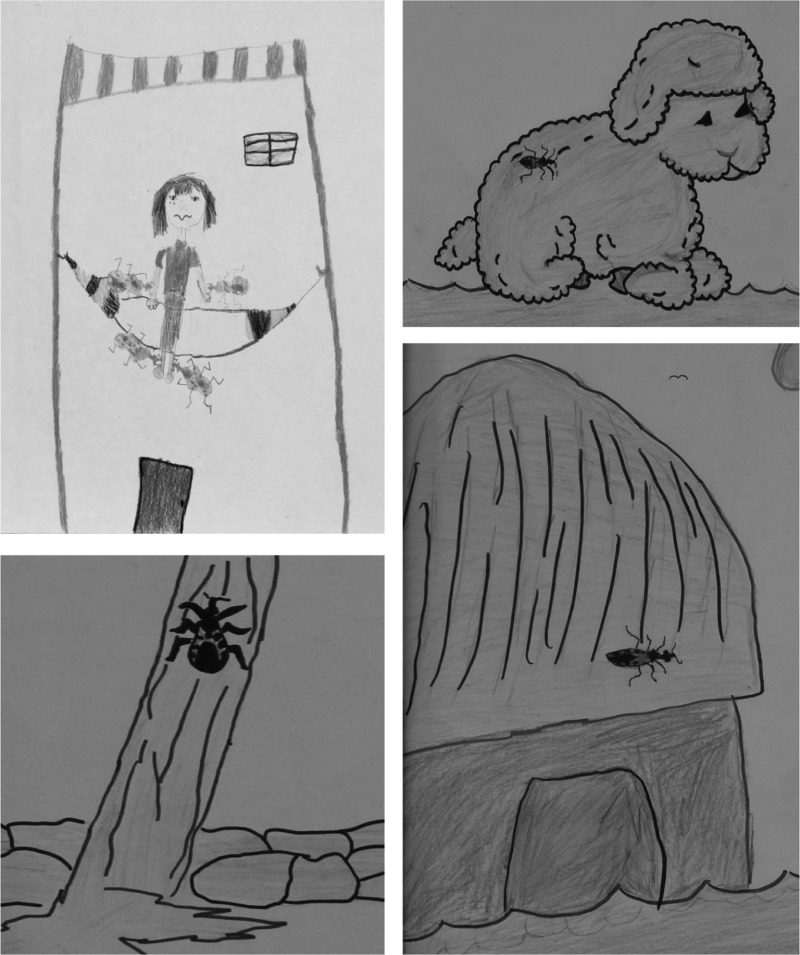
Children's perception of triatomines through drawings. Children appeared very knowledgeable about triatomines and their feeding habits, and should be engaged in education and awareness campaigns about Chagas disease and vector control.

Analysis of community members' knowledge, attitudes and perceptions of triatomines and Chagas disease indicated that both men and women were aware of and knowledgeable about *T. dimidiata*, its seasonal and nocturnal infestation patterns and blood-feeding habits.^38^ However, very few individuals related triatomines to a severe disease, and the bugs were perceived as a nuisance rather than a serious health threat. This is a barrier to community involvement in triatomine control and further community awareness on Chagas disease is needed.^38^ Through drawing contests initially organized to promote Chagas disease awareness, we also explored children's perception of triatomines and Chagas disease based on their drawings. Children were very familiar with these bugs (Figure 1), but similarly to adults, they had limited understanding of Chagas disease itself.^46^ Nonetheless, children's perceptions also reflected extensive inputs from adults, indicating that information on triatomines and Chagas disease was discussed within the entire community, thereby effectively contributing to increased awareness. Importantly, education activities specifically targeting children may be a very effective strategy to further increase community mobilization against triatomines and Chagas disease, as has been observed for malaria.^47^

In the three villages included in the study, most community-based insect control activities are currently focused on mosquitoes, which represent an important preoccupation of inhabitants. Consequently, a variety of tools are routinely used for mosquito control, the most frequently used being aerosol insecticide, mosquito coils, and plug-in repellent.^38^ Families spend an average US$32 per year for mosquito control and part of this spending could be re-directed to more effective and sustainable vector control, such as insect screens.^38^ Importantly, both men and women reported that window insect screens are highly desirable, suggesting that such a strategy would be well accepted by community members. The only significant barrier to the use of window screens was their perceived cost.

Taken together, these results provided a strong rationale for the further evaluation of window insect screens with or without improved domestic animal management in the peridomicile to reduce house infestation.

### Implementation and evaluation of the vector control intervention (Phase II)

**Figure 2.**
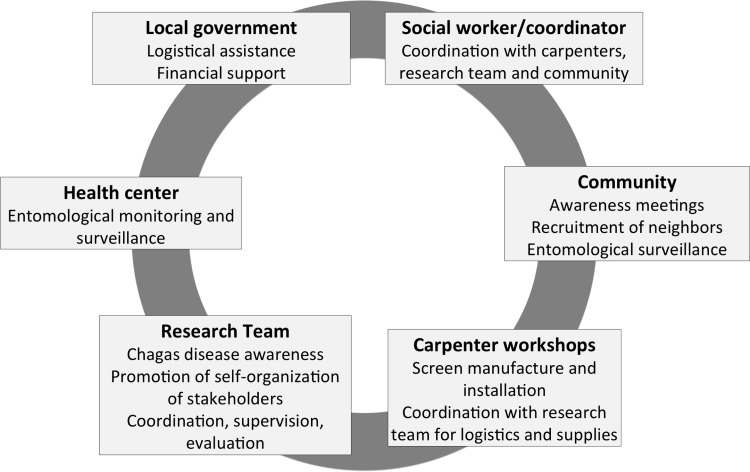
Main stakeholders and associated activities involved in the implementation of the vector control intervention. Different strategies for implementation were discussed in an iterative process over several meetings until the stakeholders reached an agreement on what was perceived as the best implementation strategy. Each stakeholder played a different role in the implementation process and was responsible for the specific activities indicated in each box.

**Figure 3.**
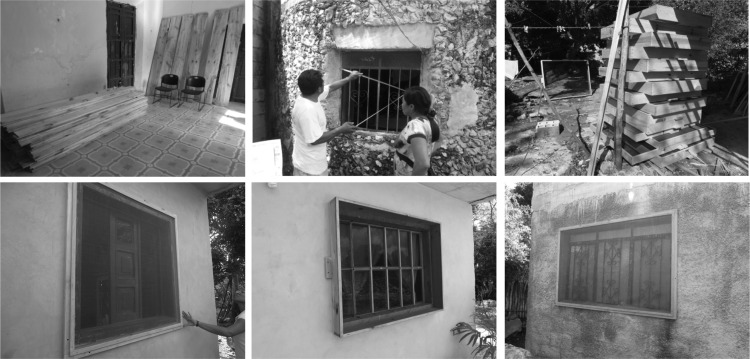
Manufacture and installation of insect screens for vector control. All screens were manufactured locally in the communities, using a high-quality fiber mesh with a wooden frame fitted to each bedroom window.

After discussions with the local government authorities and the communities, the agreed-upon strategy specified roles and responsibilities for several stakeholders (Figure 2). Since the large majority of households only have one bedroom, it was agreed that screens would be installed in the windows of a single room in each house (with two windows on average). This option was also preferred by the research team in order to: 1) respect equity between inhabitants; 2) encourage households to cover additional windows on their own, so that a positive process with a greater active implication and an increased ownership of the intervention by the community could be promoted. A social worker from each village was designated to coordinate all activities related to the implementation of the intervention. These social workers were oriented to the objectives of the project and on Chagas disease by the research group (Figure 2). Their role included: the organization of weekly community meetings at the city hall for enrollment of households; dissemination of information about Chagas disease and instructions for entomological monitoring; coordinating the distribution of materials to the carpenters and supervision of the installation of the screens; administration of a storage room provided by the local government. Carpenters were identified by the community and the local government and agreed to manufacture and install screens. The carpenters arranged for home visits to each community member to take measurements of the windows. The screen design included a high-quality fiber mesh with a wooden frame fitted to each bedroom window (Figure 3). The wood was purchased from authorized local dealers who were able to provide bulk prices and shipping to the villages. The carpenters also made arrangements with households for the delivery and installation of the screens. Once the implementation plan was in place, the research team played a minimal role in coordinating the intervention; leadership was effectively transferred to the local governments, social workers and carpenters. However, members of the research team were always present with the social workers at the meetings with the villagers, and weekly supervision was performed to evaluate community screen coverage and to arrange for delivery of materials needed by the carpenters. A total of 1606 window screens were installed in 822 households in the villages of Teya and Sudzal (Figure 3), and education about cleaning chicken coops was carried out in the village of Sudzal. The third village (Bolobá) was used as an external control of the intervention.

## Discussion

### Success and limitations of the intervention

We are currently monitoring the efficacy of this ecohealth intervention in terms of house infestation, evaluating the perceptions of the different stakeholders about the intervention, and evaluating potential changes in their awareness of triatomines and Chagas disease and in their perception of the concept of vector control.

A preliminary quantitative assessment suggests that following the intervention, the ratio of vector presence inside and outside the houses has been significantly reduced for houses with the insect screens showing, as expected, the physical barrier role played by the screens. This result is very encouraging, taking into account that only two insect screens per house were installed. A few families have also spontaneously installed additional screens on their doors or additional windows after the intervention suggesting some behavioral change. If confirmed to be effective, the evaluation of this intervention will provide a strong rationale for scaling-up to other villages in the region. The intervention also appears sustainable so far, as the large majority of screens are still in place and in perfect condition over 1.5 years after their installation. Many screens from our previous pilot study,^27^ installed 7 years ago, are also still present. However, all these preliminary results need to be confirmed to clearly establish the efficacy of the intervention.

Engaging the community and other stakeholders in Chagas disease awareness was a fundamental activity which, together with the wide acceptability of insect screens by both men and women, led to an overall high participation of all stakeholders in the proposed intervention. The inclusion of a Mayan-speaking anthropologist in the research team also facilitated access to the communities and motivated their participation. However, a greater participation of men would be needed in future projects, as our meetings were mostly attended by women while the men were working. As mentioned above, children could also be further involved to promote behavioural changes within entire families. Moreover, many adjustments to the overall implementation process had to be made by the research team to adapt to specific local situations and bypass obstacles. For example, community participation was limited in Sudzal following political tensions during local elections, as the project was perceived to be too strongly linked to the local government. Therefore we had to relocate community meetings in schools and the local health center to emphasize the non-political nature of the vector control intervention. Local governments were very supportive of the interventions, which they have readily incorporated as part of their social programs for housing improvement. For example, in Teya the mayor has agreed to fund the installation of extra window screens to large families who have additional bedrooms not covered by the project. Similarly, in Sudzal the mayor has agreed to provide paint to the villagers to protect the window screen frames and improve their durability. In both cases the research group also agreed to assist the local governments to expand the interventions.

The cost of about US$35 per house for insect screens spent in this study seems affordable for the households, even considering their modest income. The optimization of the implementation process may achieve even better prices, making scaling-up of the intervention feasible. Also, families usually spend a significant amount of their limited income on a variety of domestic insecticide products for mosquito control, even though they are well aware of the low efficacy of these products,^38^ this habit remained unchanged after the intervention. Thus, strong evidence of the efficacy of insect screens as well as further education and awareness programs are needed to motivate families to redirect this spending towards insect screens, which are often perceived as desirable but too expensive. Changes in community perception of vector control therefore needs to be promoted. Such changes not only need to reach the community, they also need to reach all stakeholders, including decision makers, vector control program personnel and local governments, so that such approaches may be incorporated into national and local guidelines to allow for scaling-up.

### Conclusions

The ecohealth approach to triatomine control followed here appears to represent a promising alternative to vertically-organized insecticide spraying, allowing for greater community participation and ownership of the control intervention, as well as a potentially more sustainable control of intrusive triatomines in the region. Importantly, the proposed intervention is expected not only to prevent Chagas disease transmission, but also contribute to the prevention of other vector-borne diseases such as dengue fever, since physical barriers will also reduce mosquito abundance in the houses. Cleaner backyards and a reduced incidence of vector-borne diseases are also expected to lead to an improvement of the overall living conditions and quality of life of the communities, bringing them additional benefits beyond Chagas disease prevention, as mentioned earlier in similar ecohealth interventions.^48^
